# Elective primary total hip arthroplasty in rheumatoid arthritis versus osteoarthritis: in-hospital outcomes from the national inpatient sample, 2016–2021

**DOI:** 10.1007/s00402-026-06523-w

**Published:** 2026-10-07

**Authors:** Binyamin Finkel, Hadar Gan-Or, Shaked Ankol, Mohamd Tawatha, Eyal Behrbalk

**Affiliations:** 1 Department of Orthopedic Surgery, Hillel Yaffe Medical Center, Hadera, Israel; 2 Ruth and Bruce Rappaport Faculty of Medicine, Technion-Israel Institute of Technology, Haifa, Israel

**Keywords:** Total hip arthroplasty; Rheumatoid arthritis; Osteoarthritis; In-hospital outcomes; Postoperative complications; National Inpatient Sample

## Abstract

**Background:**

Whether coded rheumatoid arthritis (RA) among patients undergoing elective total hip arthroplasty (THA) is associated with excess short-term inpatient morbidity in contemporary U.S. practice remains uncertain.

**Methods:**

We performed a retrospective cohort study using the National Inpatient Sample (2016–2021). Adults undergoing elective primary THA were identified using ICD-10-CM/PCS codes. The exposure cohort included admissions with coded RA in any diagnosis position. The comparator cohort included admissions with narrow primary hip osteoarthritis (OA) as the principal diagnosis and no RA. Propensity scores were estimated from demographic, socioeconomic, hospital, and comorbidity variables. Inverse probability of treatment weighting (IPTW) with survey weighting was used for the primary analysis, followed by doubly adjusted regression models that included age and all variables with persistent post-weighting imbalance greater than 0.10 (female sex, osteoporosis, chronic obstructive pulmonary disease, chronic anemia, depression, and race). Additional sensitivity analyses included stricter weight truncation, a restriction analysis, conventional multivariable modeling, and sex-stratified analyses addressing residual sex imbalance.

**Results:**

A total of 274,582 admissions met eligibility criteria; 274,539 were included in the final analytic cohort after exclusion of 43 records with missing or invalid propensity-weight inputs. The analytic cohort included 7,264 RA admissions and 267,275 OA admissions. In the primary weighted analysis, RA was associated with higher odds of acute blood loss anemia (OR 1.30, 95% CI 1.23–1.38), red blood cell transfusion (OR 1.40, 95% CI 1.23–1.60), acute kidney injury (OR 1.47, 95% CI 1.26–1.71), non-home discharge (OR 1.27, 95% CI 1.19–1.36), and a composite hematologic/renal in-hospital complication (OR 1.31, 95% CI 1.24–1.39).RA was also associated with slightly longer length of stay (+ 0.14 days, 95% CI + 0.10 to + 0.19) and modestly higher total hospital charges (+$1,018, 95% CI +$89 to +$1,947). Findings were directionally consistent across sensitivity analyses, including stricter weight truncation, restriction to admissions with principal OA plus concomitant RA, and sex-stratified analyses.

**Conclusions:**

In this contemporary national THA cohort, coded RA was associated with a small but consistent increase in in-hospital complication and resource-utilization burden compared with OA. These findings should be interpreted as adjusted associations rather than causal effects, and the modest effect sizes should not be overstated. They may provide context for perioperative risk assessment and discharge planning among patients with coded RA undergoing elective THA.

## Introduction

 Rheumatoid arthritis is a chronic systemic inflammatory disease in which improved pharmacologic control has reduced structural joint damage and altered the natural history of end-stage joint destruction [1,0.2]. Even so, major weight-bearing joints remain vulnerable, and total hip arthroplasty (THA) continues to play an important role for pain relief and functional restoration in patients with advanced hip involvement [1–4].

At the population level, the incidence of arthroplasty in RA has fallen in the biologic era, yet the absolute need for THA has not disappeared [3, 4]. Patients with RA who present for arthroplasty may still differ meaningfully from patients with osteoarthritis (OA) because of chronic inflammation, glucocorticoid exposure, immunomodulatory therapy, poorer bone quality, anemia, and a higher burden of systemic comorbidity [1, 5–8].

Prior arthroplasty literature suggests that RA may be associated with excess postoperative morbidity, including infection, dislocation, transfusion, readmission, and worse patient-reported outcomes [9–18]. However, much of this evidence either combines hip and knee arthroplasty, reflects earlier treatment eras, focuses on mid-term complications rather than the index hospitalization, or arises from single-country registries rather than contemporary U.S. national inpatient data [9–18].

Less is known about whether coded RA among elective THA recipients is associated with immediate inpatient outcomes that may affect perioperative management and discharge planning, including acute blood loss anemia, transfusion, AKI, discharge disposition, length of stay, and hospital charges. These outcomes are distinct from longer-term infection, revision, readmission, instability, and patient-reported outcomes, and may be particularly sensitive to comorbidity burden, perioperative blood management, renal vulnerability, and discharge pathways. Accordingly, contemporary U.S. THA-specific inpatient data comparing admissions with coded RA and narrow primary OA remain limited, leaving uncertainty regarding immediate in-hospital risk in modern practice. We therefore used the National Inpatient Sample to compare elective primary THA admissions with coded RA versus narrow OA admissions during 2016–2021, using survey-weighted propensity methods and multiple sensitivity analyses. We hypothesized that admissions with coded RA would be associated with modestly higher in-hospital complication rates, greater discharge complexity, and greater resource use despite modern practice [3, 5, 9, 11, 13, 16, 17].

## Methods

### Data source and study design

We performed a retrospective cohort study using the National Inpatient Sample (NIS) for calendar years 2016 through 2021. The NIS is a 20% stratified sample of U.S. inpatient discharges and allows generation of national estimates when survey discharge weights are applied. Because the dataset is de-identified and publicly available, institutional review board approval was not required.

### Study population

We identified adult admissions (age ≥ 18 years) undergoing elective primary THA using ICD-10-PCS procedure codes in PR1. The RA cohort required a qualifying RA diagnosis in any diagnosis field (DX1–DX40), capturing coded RA among elective THA admissions rather than restricting the exposure to RA as the principal surgical indication. This definition was selected to evaluate the clinically relevant perioperative phenotype of patients with coded RA undergoing THA, regardless of whether RA was recorded as the principal indication for surgery. Therefore, the exposure should be interpreted as coded RA among patients undergoing elective primary THA, rather than THA performed specifically for RA-related hip disease. The OA cohort required narrow primary hip OA as the principal diagnosis (DX1 only) and no RA diagnosis. Exclusion criteria were applied to remove fracture, revision or failed arthroplasty, periprosthetic pathology, infection, malignancy, osteonecrosis, other inflammatory arthritides, dysplasia/post-traumatic arthropathy, hemiarthroplasty, resurfacing, and revision-related procedures. The full coding framework is provided in Appendix Table 4.

### Covariates

Baseline covariates entered into the propensity score model included age, sex, race/ethnicity, primary payer, ZIP-code income quartile, hospital region, hospital location/teaching status, hospital bed size, year, transfer-in status, and comorbidities listed in Appendix Table 5.

### Outcomes

The prespecified primary binary outcomes were acute blood loss anemia, red blood cell (RBC) transfusion, acute kidney injury (AKI), non-home discharge, and a composite hematologic/renal in-hospital complication composed of acute blood loss anemia, RBC transfusion, and AKI. Continuous outcomes were length of stay and total hospital charges. Rare outcomes (mortality, deep venous thrombosis, pulmonary embolism, pneumonia, sepsis, acute myocardial infarction, wound infection, and a hip-dislocation proxy code) were summarized descriptively because events within the RA cohort were sparse and regression estimates were likely to be unstable.

### Statistical analysis

Baseline characteristics were summarized with means or percentages and standardized mean differences (SMDs). A propensity score for RA exposure was estimated using the covariates listed in Appendix Table 5. Inverse probability of treatment weights targeting the average treatment effect were generated and winsorized at the 1st and 99th percentiles for the primary analysis. Survey discharge weights supplied by HCUP were incorporated in all outcome models. Binary outcomes were analyzed with survey-weighted logistic regression and continuous outcomes with survey-weighted linear regression. Because residual post-weighting imbalance remained for female sex, osteoporosis, chronic obstructive pulmonary disease, chronic anemia, depression, and race, the primary doubly adjusted models included age and all variables with persistent post-weighting imbalance. Sensitivity analyses included: (1) pure IPTW models without additional covariate adjustment; (2) stricter 5th/95th percentile weight truncation; (3) conventional multivariable models using DISCWT only and all propensity-score covariates; and (4) a restriction analysis limited to admissions with principal OA and concomitant RA coded in a non-principal diagnosis field. To address the residual imbalance in sex, we additionally performed a post hoc sex-stratified sensitivity analysis. The analytic cohort was stratified into female-only and male-only admissions. Within each sex stratum, the propensity score was re-estimated using the same covariate framework as the primary analysis, excluding sex because it was constant within stratum. IPTW was generated and winsorized at the 1st and 99th percentiles, survey discharge weights were incorporated, and outcome models were repeated within each stratum with additional adjustment for covariates with persistent post-weighting imbalance greater than 0.10. This stratified analysis was intended as a robustness assessment addressing residual sex imbalance rather than as a formal test of RA-by-sex effect modification. Calendar year was included in the propensity-score framework to account for secular changes across 2016–2021. Two-sided p values < 0.05 were considered statistically significant.

## Results

### Cohort assembly

A total of 274,582 admissions met eligibility criteria, including 7,265 RA admissions and 267,317 OA admissions. After exclusion of 43 records with missing or invalid propensity-weight inputs, the final analytic cohort included 274,539 admissions: 7,264 with RA and 267,275 with OA.

### Baseline characteristics

Before weighting, the RA group was similar in age but more often female and carried a higher burden of osteoporosis, chronic anemia, chronic obstructive pulmonary disease, depression, and several medical comorbidities. Post-weighting balance improved substantially for most variables, although modest residual imbalance persisted for female sex, osteoporosis, chronic anemia, chronic obstructive pulmonary disease, depression, and overall race distribution (Table 1; Appendix Table 7).

### Primary weighted analysis

In the primary doubly adjusted IPTW analysis, RA was associated with higher odds of acute blood loss anemia (OR 1.30, 95% CI 1.23–1.38), RBC transfusion (OR 1.40, 95% CI 1.23–1.60), AKI (OR 1.47, 95% CI 1.26–1.71), non-home discharge (OR 1.27, 95% CI 1.19–1.36), and a composite hematologic/renal in-hospital complication (OR 1.31, 95% CI 1.24–1.39). RA was also associated with longer length of stay (+ 0.14 days, 95% CI + 0.10 to + 0.19) and higher total hospital charges (+$1,018, 95% CI +$89 to +$1,947) (Table 2) . Group-specific IPTW-weighted means for length of stay and charges are also presented in Table 2.

### Sensitivity analyses

The direction and overall magnitude of association remained broadly similar in models without additional covariate adjustment, in models with stricter 5th/95th percentile weight truncation, and in conventional multivariable regression. In the restriction analysis comparing admissions with principal OA plus concomitant RA (*n* = 6,610) versus OA-only admissions (*n* = 267,275), the excess risk persisted for acute blood loss anemia (OR 1.27), RBC transfusion (OR 1.20), AKI (OR 1.42), non-home discharge (OR 1.20), and a composite hematologic/renal in-hospital complication (OR 1.27). Length of stay remained longer (+ 0.11 days), whereas the difference in total hospital charges was no longer statistically significant (*p* = 0.14) (Table 3).

In the sex-stratified sensitivity analysis, findings remained directionally consistent within sex strata (Appendix Table 8). The female-only analysis included 152,877 admissions (5,320 RA and 147,557 OA), and the male-only analysis included 121,662 admissions (1,944 RA and 119,718 OA). Among female admissions, RA remained associated with higher odds of acute blood loss anemia (OR 1.34, 95% CI 1.26–1.43), RBC transfusion (OR 1.50, 95% CI 1.31–1.73), AKI (OR 1.58, 95% CI 1.31–1.91), non-home discharge (OR 1.32, 95% CI 1.23–1.42), and the composite hematologic/renal in-hospital complication (OR 1.36, 95% CI 1.27–1.45), as well as longer length of stay (+ 0.19 days, 95% CI + 0.14 to + 0.25) and higher total hospital charges (+$1,705, 95% CI +$651 to +$2,759). Among male admissions, estimates were directionally consistent for acute blood loss anemia (OR 1.15, 95% CI 1.01–1.30), AKI (OR 1.46, 95% CI 1.13–1.88), non-home discharge (OR 1.31, 95% CI 1.14–1.51), the composite outcome (OR 1.19, 95% CI 1.05–1.33), and length of stay (+ 0.09 days, 95% CI + 0.03 to + 0.16), although the associations with RBC transfusion and total hospital charges were not statistically significant. These findings suggest that the primary results were not driven solely by the higher proportion of female patients in the RA cohort.

### Rare outcomes

Rare events were uncommon and were therefore summarized descriptively. Unadjusted survey-weighted descriptive rates were numerically higher in RA for deep venous thrombosis (0.14% vs. 0.07%), pulmonary embolism (0.16% vs. 0.07%), pneumonia (0.23% vs. 0.14%), sepsis (0.08% vs. 0.05%), and acute myocardial infarction (0.11% vs. 0.09%). In-hospital mortality (0.00% vs. 0.03%), wound infection (0.01% vs. 0.02%), and the exploratory hip-dislocation proxy outcome (< 0.01% in both groups) were very infrequent. No inferential comparisons were performed for these rare endpoints; these descriptive comparisons are hypothesis-generating only and should not be interpreted as adjusted risk estimates.

## Discussion

The primary finding of this study is that, in a contemporary national cohort of elective primary THA, coded RA was associated with modestly higher odds of acute blood loss anemia, RBC transfusion, AKI, non-home discharge, and a composite hematologic/renal in-hospital complication. Because RA was defined by a diagnosis code in any diagnosis position rather than as the principal surgical indication, these associations reflect outcomes among THA recipients with coded RA rather than a phenotype selected specifically for RA-driven arthroplasty. The direction of association was consistent across several analytic approaches, but the absolute differences were generally small and should be interpreted in the context of the large sample size and administrative design.

These findings are broadly concordant with earlier literature showing that patients with RA undergoing arthroplasty may face a more complex perioperative course than patients with OA [9–18]. Prior systematic reviews and registry studies have linked RA with higher risks of medical complications, transfusion, readmission, and, in some reports, infection or instability after total joint arthroplasty [10–18]. Several of those studies, however, combined hip and knee procedures or emphasized longer-term outcomes rather than the index hospitalization. Our study extends that literature by focusing specifically on contemporary elective primary THA in the ICD-10 era and by evaluating immediate in-hospital outcomes that are directly relevant to perioperative planning, including hematologic/renal complications, discharge disposition, length of stay, and hospital charges.

The sex-stratified sensitivity analysis directly addressed the persistent imbalance in sex after IPTW. The directionally consistent associations observed within female-only and male-only strata suggest that the primary findings were not explained solely by the higher proportion of female patients in the RA cohort. At the same time, some male-stratified estimates, particularly for RBC transfusion and hospital charges, were less precise, which is expected given the smaller number of male RA admissions and supports cautious interpretation of subgroup-specific effects.

Residual imbalance after IPTW should also be considered when interpreting these findings. Although weighting improved balance for most measured covariates, imbalance persisted for several clinically relevant variables, including sex, osteoporosis, chronic anemia, COPD, depression, and race. This persistent imbalance indicates incomplete comparability between the RA and OA groups on measured baseline characteristics and may also reflect broader clinical differences that are not fully captured in the NIS. The primary models therefore used doubly adjusted regression including these variables, and the sex-stratified analysis provided an additional targeted assessment of the largest residual imbalance. Nevertheless, these approaches reduce but cannot eliminate residual confounding, including confounding from unmeasured factors, and the estimates should be interpreted as adjusted associations rather than causal effects.

Although inverse probability of treatment weighting (IPTW) was intentionally selected because it preserves the full study population while estimating the average treatment effect, alternative propensity-score approaches such as overlap weighting or propensity-score matching may achieve improved covariate balance in certain settings. However, these approaches estimate different target populations and may reduce sample size or generalizability. Future studies applying complementary causal inference methods could further evaluate the robustness of the present findings.

The observed associations with acute blood loss anemia and transfusion may reflect unmeasured differences in baseline hemoglobin, inflammatory burden, nutritional status, frailty, glucocorticoid exposure, bone quality, intraoperative blood loss, or perioperative blood-management practices [1–8, 14]. Similarly, the higher odds of AKI may relate to unmeasured differences in baseline renal function, perioperative hemodynamics, fluid management, nephrotoxic exposure, or medication use [5–8, 16]. Because the NIS does not contain laboratory values, medication exposure, intraoperative data, or detailed perioperative management variables, none of these mechanisms could be tested directly. They should therefore be regarded strictly as hypothesis-generating possibilities rather than explanations established by the present dataset.

From a practical standpoint, although the observed associations were statistically significant, the absolute differences in event rates were generally modest and should therefore be interpreted in the context of their clinical relevance. These findings may provide context for perioperative planning, but their clinical magnitude should not be overstated. The adjusted 0.14-day difference in length of stay is unlikely, by itself, to materially alter discharge planning. Likewise, the $1,018 difference in hospital charges was modest relative to overall charges, may fall within inter-hospital billing variation, and should not be interpreted as an equivalent difference in true cost; moreover, the charge difference was no longer statistically significant in the restriction analysis. Overall, the results are best interpreted as evidence of a small but consistent increase in inpatient burden among patients with coded RA, rather than a large difference in perioperative risk.

Our descriptive rare-event analyses should be interpreted cautiously. Although several rare complications were numerically more frequent in RA, absolute event rates were low, no adjusted or inferential comparisons were performed for these endpoints, and the study was not designed to support definitive inference for them. In particular, the hip-dislocation variable should be viewed only as an exploratory administrative proxy rather than a definitive prosthetic dislocation endpoint. Rare administrative outcomes therefore should not drive interpretation of the study.

### Strengths

This study has several strengths. First, it uses a large contemporary U.S. national dataset and focuses specifically on elective primary THA rather than combining hip and knee arthroplasty. Second, it applies survey-weighted propensity methods with multiple sensitivity analyses, including a clinically useful restriction analysis. Third, it addresses outcomes that are immediately relevant to perioperative planning and discharge disposition.

### Limitations

This study also has important limitations. First, diagnoses, procedures, comorbidities, and complications were identified using administrative codes. Coding of outcomes such as acute blood loss anemia and AKI may vary across hospitals, coders, and calendar years, and administrative data may be subject to undercoding, overcoding, or changes in coding practices over time. Differential coding practices between patients with coded RA and OA cannot be excluded and could contribute to some of the observed associations. The NIS does not allow confirmation of laboratory thresholds, clinical severity, timing of onset, or whether a coded complication was directly attributable to the surgical episode. Second, the NIS does not capture RA disease activity, serologic status, glucocorticoid dose, biologic or targeted synthetic DMARD exposure, baseline hemoglobin, baseline creatinine, nutritional status, blood-management protocols, surgical approach, implant design, intraoperative blood loss, perioperative fluid management, nephrotoxic exposure, or post-discharge outcomes. Third, the analysis is limited to the index hospitalization and therefore cannot assess longer-term revision, infection, readmission, prosthetic instability, or functional recovery. Rare outcomes were too sparse for stable multivariable modeling, and the exploratory hip-dislocation proxy should be interpreted with particular caution. Fourth, total hospital charges are not equivalent to true cost and may be influenced by hospital billing practices, regional pricing, and secular changes. Fifth, the exposure definition captures coded RA among elective THA admissions and therefore does not isolate RA as the principal surgical indication in every case; the findings should be interpreted as outcomes among patients with coded RA undergoing THA rather than outcomes of THA performed specifically for RA-related hip disease. Sixth, the study period included the COVID-19 pandemic, which may have influenced elective THA case selection, length of stay, discharge to rehabilitation or skilled nursing facilities, and hospital charges during 2020–2021. Although calendar year was included in the propensity-score framework to account for secular trends, year adjustment may not fully capture pandemic-specific changes. We did not perform a dedicated 2016–2019 versus 2020–2021 period-stratified analysis; therefore, the present study cannot determine whether the association between coded RA and inpatient outcomes differed during the pandemic period, and residual pandemic-related changes in perioperative practice and discharge pathways may remain. Finally, although weighting improved balance substantially and the primary models incorporated full double adjustment, meaningful residual confounding remains possible because post-weighting imbalance persisted for several variables, particularly sex, osteoporosis, chronic anemia, chronic obstructive pulmonary disease, depression, and race.

## Conclusion

In a contemporary national sample of elective primary THA admissions, coded RA was associated with a small but consistent increase in in-hospital complication burden, discharge complexity, length of stay, and hospital charges compared with OA. These findings do not establish causality and should be interpreted in light of residual confounding, administrative-coding limitations, and the modest absolute effect sizes. They may provide context for perioperative risk assessment and discharge planning among patients with coded RA undergoing elective THA (see Tables 1 and 2).

**Table 1:** Selected baseline characteristics of the final analytic cohort

Variable	OA (*n* = 267,275)	RA (*n* = 7,264)	Pre-IPTW SMD	Post-IPTW SMD
Age, mean years	66.5	66.6	0.014	0.028
Female sex, %	55.0	73.0	0.383	0.270†
White race, %	87.0	81.3	0.167	0.112†
Hypertension, %	60.4	64.0	0.067	0.048
Diabetes mellitus, %	15.7	17.0	0.039	< 0.10
Chronic kidney disease, %	6.5	8.0	0.056	< 0.10
COPD, %	6.8	12.0	0.173	0.126†
Congestive heart failure, %	2.7	4.0	0.093	< 0.10
Coronary artery disease, %	11.3	13.0	0.065	< 0.10
Chronic anemia, %	7.7	12.0	0.161	0.109†
Osteoporosis, %	4.0	11.0	0.252	0.181†
Obesity, %	24.0	25.0	0.023	0.016
Depression, %	12.4	18.0	0.166	0.112†

†Residual post-IPTW imbalance > 0.10. Percentages are rounded to one decimal

**Table 2:** Primary weighted outcomes (Analysis 1) and stricter winsorization sensitivity analysis (Analysis 3)

Outcome	OA IPTW-weighted rate/mean	RA IPTW-weighted rate/mean	Primary adjusted OR/coefficient (95% CI)	Sensitivity A3 OR/coefficient (95% CI)	*p* value
*Binary outcomes—adjusted odds ratio (95% CI)*					
Acute blood loss anemia	16.6%	21.6%	1.30 (1.23–1.38)	1.32 (1.24–1.40)	< 0.001
RBC transfusion	2.1%	3.5%	1.40 (1.23–1.60)	1.41 (1.24–1.60)	< 0.001
Acute kidney injury	1.7%	2.5%	1.47 (1.26–1.71)	1.48 (1.27–1.72)	< 0.001
Non-home discharge	13.4%	18.2%	1.27 (1.19–1.36)	1.29 (1.20–1.37)	< 0.001
Composite hematologic/renal in-hospital complication	18.2%	23.8%	1.31 (1.24–1.39)	1.33 (1.25–1.40)	< 0.001
*Continuous outcomes—adjusted mean difference (95% CI)*					
Length of stay, days	1.91	2.13	+ 0.14 (+ 0.10 to + 0.19)	+ 0.15 (+ 0.11 to + 0.20)	< 0.001
Total hospital charges, USD	$62,070	$63,870	+$1,018 (+$89 to +$1,947)	+$1,152 (+$215 to +$2,089)	0.032

Primary models used survey-weighted IPTW with 1st/99th percentile winsorization and doubly adjusted regression including age, female sex, osteoporosis, COPD, chronic anemia, depression, and race. Analysis 3 used 5th/95th percentile winsorization with the same covariate adjustment. For continuous outcomes, the OA and RA columns report descriptive group-specific IPTW-weighted means obtained from the primary survey design. These descriptive means are presented separately from the adjusted between-group regression coefficient and are not expected to equal its arithmetic contrast (see Table 3).

**Table 3:** Triangulation analysis (Analysis 5): principal OA with concomitant RA versus OA only

Outcome	OA-only rate	OA + RA rate	OR / coefficient (95% CI)	*p* value
Acute blood loss anemia	16.6%	21.1%	1.27 (1.19–1.35)	< 0.001
RBC transfusion	2.1%	3.3%	1.20 (1.04–1.38)	0.014
Acute kidney injury	1.7%	2.4%	1.42 (1.21–1.67)	< 0.001
Non-home discharge	13.4%	17.8%	1.20 (1.12–1.29)	< 0.001
Composite hematologic/renal in-hospital complication	18.2%	23.0%	1.27 (1.19–1.34)	< 0.001
Length of stay, days	–	–	+ 0.11 (+ 0.06 to + 0.15)	< 0.001
Total hospital charges, USD	–	–	+$709 (-$237 to +$1,655)	0.14

Analysis 5 compared admissions with principal OA and concomitant RA coded in a non-principal diagnosis field (*n* = 6,610) with OA-only admissions (*n* = 267,275). Models used the same doubly adjusted IPTW framework as the primary analysis, including age, female sex, osteoporosis, COPD, chronic anemia, depression, and race.

Values are odds ratios for binary outcomes and regression coefficients for continuous outcomes. The female-only analysis included 152,877 admissions (5,320 RA and 147,557 OA), and the male-only analysis included 121,662 admissions (1,944 RA and 119,718 OA). Sex-stratified sensitivity analyses were performed separately among female-only and male-only admissions. Propensity scores were re-estimated within each sex stratum using the same covariate framework as the primary analysis, excluding sex. IPTW was winsorized at the 1st and 99th percentiles and combined with NIS survey discharge weights. Outcome models used survey-weighted regression with additional adjustment for residual post-weighting imbalance. Female-only models were additionally adjusted for age and osteoporosis. Male-only models were additionally adjusted for age, COPD, congestive heart failure, coronary artery disease, osteoporosis, depression, and Hispanic race. This analysis was intended to assess robustness to residual sex imbalance and was not designed as a formal test of RA-by-sex effect modification.

## Data Availability

The National Inpatient Sample data used in this study are available from the Healthcare Cost and Utilization Project under a data-use agreement. Because of HCUP data-use restrictions, the authors cannot publicly share or redistribute the raw data. Study-specific ICD-10-CM/PCS code lists are provided in the Appendix, and analytic code is available from the corresponding author upon reasonable request, subject to applicable data-use restrictions.

## Appendix

See Tables 4, 5, 6, 7, 8.

**Table 4:** Coding framework used for cohort assembly and outcomes

Category	ICD-10-CM/PCS code(s)	Notes
Inclusion—RA	M05.*, M060, M062*, M063*, M068*, M069	RA diagnosis in any diagnosis field (DX1–DX40).
Inclusion—OA	M16.0, M16.1*, M16.9	Narrow primary hip OA; required in DX1 for the OA cohort.
Procedure—elective primary THA (PR1)	0SR901*, 0SR902*, 0SR903*, 0SR904*, 0SR90J*, 0SRB01*, 0SRB02*, 0SRB03*, 0SRB04*, 0SRB0J*	Right- and left-sided primary THA code families, including fixation/material variants.
Exclude—fracture diagnoses	S72.*, S32.4*, S32.5*, S32.7*	Hip or pelvic fracture.
Exclude—prosthetic/mechanical complications	T84.0*, T84.1*, T84.5*, T84.8*, T84.9*	Mechanical complication or infection of prosthesis.
Exclude—periprosthetic fracture	M97.0*	Periprosthetic fracture; excluded because present-on-admission status is not reliably available in NIS for this use.
Exclude—malignancy or pathologic fracture	C40.*, C41.*, C79.5*, C80.0, M84.5*	Bone malignancy, metastasis, or pathologic fracture context.
Exclude—osteonecrosis	M87.*	Avascular necrosis.
Exclude—other inflammatory arthritides	M07.*, L40.5, M08.*, M45.*, M32.*	Psoriatic, juvenile, ankylosing, lupus-related arthropathy.
Exclude—misc. non-target diagnoses	M06.1, M06.4, Z96.64*, M16.4*, M16.5*, Q65.*, M24.7*	Still disease, other inflammatory polyarthropathy, prior arthroplasty status, post-traumatic OA, dysplasia, other joint disorders.
Exclude—musculoskeletal infection	M00.*, M01.*, M86.*	Septic arthritis or osteomyelitis.
Exclude—hemiarthroplasty	0SRR0*, 0SRS0*	Partial hip replacement / hemiarthroplasty in PR1.
Exclude— resurfacing	0SU90*, 0SUB0*	Hip resurfacing in PR1.
Exclude— removal or revision-related procedures	0SW9*, 0SWB*, 0SP9*, 0SPB*	Revision, removal, or component-related procedures.
Exclude—tissue-substitute THA codes	0SR907*, 0SR90K*, 0SRB07*, 0SRB0K*	Autologous/nonautologous tissue substitute code families; excluded from the primary THA construct.
Outcome—blood loss anemia	D62	Acute posthemorrhagic anemia.
Outcome—RBC transfusion	30233N1, 30243N1, 30233P1, 30243P1	Packed RBC transfusion procedure codes.
Outcome—AKI	N17.*	Acute kidney injury.
Outcome—non-home discharge	DISPUNIFORM = 2 or 5	Transfer, skilled nursing, or other facility discharge.
Rare outcomes (descriptive only)	I82.4*, I26.*, J12–J18, A40–A41, I21.*, M24.35*	DVT, PE, pneumonia, sepsis, AMI, and an exploratory hip-dislocation proxy code. The hip-dislocation proxy should be interpreted cautiously.

An asterisk (*) denotes an ICD-10-CM/PCS code-family wildcard

**Table 5:** Propensity-score covariate framework

Variable category	Variables included	Notes
Demographics	Age (continuous), female sex	
Race/ethnicity	Black, Hispanic, Asian/Other, missing (reference: White)	Four dummy variables.
Hospital region	Midwest, South, West (reference: Northeast)	Three dummy variables.
Hospital location/teaching	Urban non-teaching, urban teaching (reference: rural)	
Hospital bed size	Medium, large (reference: small)	
Primary payer	Medicaid, private, other (reference: Medicare)	
ZIP income quartile	Q2, Q3, Q4 (reference: Q1)	Area-level socioeconomic proxy.
Year	2017–2021 (reference: 2016)	Accounts for temporal trends.
Transfer in	TRAN_IN = 1	
Comorbidities	HTN, diabetes, CKD, COPD, CHF, CAD, dyslipidemia, chronic anemia, IBD, OSA, osteoporosis, obesity, coagulation disorder, smoking, depression, anxiety	Binary variables.

**Table 6:** Analytic plan and sensitivity analyses

Analysis	Method	Covariates	Purpose
A1 (primary)	Survey-weighted IPTW, winsorized 1st/99th percentiles, doubly adjusted regression	Age, female sex, osteoporosis, COPD, chronic anemia, depression, and race	Primary estimate (ATE estimand).
A2	Survey-weighted IPTW only	Treatment indicator only	Sensitivity analysis without additional adjustment.
A3	Survey-weighted IPTW, winsorized 5th/95th percentiles, doubly adjusted regression	Age, female sex, osteoporosis, COPD, chronic anemia, depression, and race	Sensitivity analysis with stricter truncation.
A4	Survey-weighted multivariable regression using DISCWT only	All propensity-score covariates	Sensitivity analysis using conventional multivariable adjustment.
A5	Survey-weighted IPTW with same doubly adjusted framework	Age, female sex, osteoporosis, COPD, chronic anemia, depression, and race	Triangulation: principal OA plus concomitant RA vs. OA only.
A6	Post hoc survey-weighted IPTW repeated separately within female-only and male-only strata	Same propensity-score covariate framework, excluding sex; doubly adjusted for residual post-IPTW imbalance within each stratum	Post hoc sensitivity analysis addressing residual sex imbalance.

**Table 7:** Key balance diagnostics before and after IPTW

Variable	OA	RA	Pre-IPTW SMD	Post-IPTW SMD
Age (mean)	66.5	66.6	0.014	0.028
Female sex	55%	73%	0.383	0.270†
COPD	6.8%	12.0%	0.173	0.126†
Chronic anemia	7.7%	12.0%	0.161	0.109†
Osteoporosis	4.0%	11.0%	0.252	0.181†
Depression	12.4%	18.0%	0.166	0.112†
Anxiety	12.6%	17.0%	0.119	0.081
Race (overall)	—	—	0.167	0.112†
Hypertension	60.4%	64.0%	0.067	0.048
Dyslipidemia	43.8%	43.0%	0.025	0.015
Obesity	24.0%	25.0%	0.023	0.016
Smoking	9.1%	10.0%	0.020	0.012

†Post-IPTW SMD > 0.10

**Table 8:** Sex-stratified sensitivity analysis of in-hospital outcomes after elective primary THA in RA versus OA

Outcome	Female-only estimate (95% CI)	*p* value	Male-only estimate (95% CI)	*p* value
Acute blood loss anemia	OR 1.34 (1.26–1.43)	< 0.001	OR 1.15 (1.01–1.30)	0.031
RBC transfusion	OR 1.50 (1.31–1.73)	< 0.001	OR 1.26 (0.89–1.77)	0.193
Acute kidney injury	OR 1.58 (1.31–1.91)	< 0.001	OR 1.46 (1.13–1.88)	0.004
Non-home discharge	OR 1.32 (1.23–1.42)	< 0.001	OR 1.31 (1.14–1.51)	< 0.001
Composite hematologic/renal in-hospital complication	OR 1.36 (1.27–1.45)	< 0.001	OR 1.19 (1.05–1.33)	0.005
Length of stay, days	+ 0.19 (+ 0.14 to + 0.25)	< 0.001	+ 0.09 (+ 0.03 to + 0.16)	0.005
Total hospital charges, USD	+$1,705 (+$651 to +$2,759)	0.002	+$291 (−$1,347 to +$1,928)	0.728
